# A tricky case of unilateral orbital inflammation: carotid cavernous fistula in Graves-Basedow disease

**DOI:** 10.22336/rjo.2021.40

**Published:** 2021

**Authors:** Javier Lacorzana, Carlos Rocha-de-Lossada, Santiago Ortiz-Perez

**Affiliations:** * Department of Ophthalmology, Virgen de las Nieves University Hospital, Granada, Spain; ** Doctoral Programme in Clinical Medicine and Public Health, University of Granada, Granada, Spain

**Keywords:** Graves-Basedow disease, Graves’ ophthalmopathy, Carotid Cavernous Fistula, orbit, orbital inflammation

## Abstract

**Purpose:** To describe a rare clinical case of Carotid Cavernous Fistula (CCF) in Graves-Basedow disease (GBD).

**Method:** A 62-year-old female with history of GBD and inactive Graves’ ophthalmopathy (GO) was admitted with progressive exophthalmos in her right eye (RE) and diagnosed with GO reactivation the previous month.

**Results:** On examination, dilated and tortuous conjunctival blood vessels, chemosis and exophthalmos were observed in the RE. There was an asymmetry in the intraocular pressures of 20 mmHg in the RE and 10 mmHg in the LE. Laboratory results showed normal thyroid function and positivity of some of the antibodies related to immune thyroid disorders. Neuroimaging showed an early and abnormal filling of the cavernous sinus (CS) and an enlarged superior ophthalmic vein. Consequently, the diagnosis of CCF was established. Interventional treatment was performed with good clinical outcome and no recurrence after 6 months of follow-up.

**Conclusions:** CCFs are abnormal arteriovenous connections between the carotid arteries and CS. CCF picture can mimic the GO’s manifestations. Thus, CCF should be considered in the differential diagnosis of GO, especially in unilateral, asymmetric, and atypical cases. We reported herein a case of CCF in a patient diagnosed with GBD, having a previous history of inactive GO, a diagnostic challenge, since the first presumed diagnosis in patients with GBD is always GO. To the best of our knowledge, there are only three previous reports in the medical literature in which the CCF was diagnosed in a GBD patient with a history of GO.

**Abbreviations**:

CCF = Carotid Cavernous Fistula, GBD = Graves-Basedow disease, GO = Graves’ ophthalmopathy, CS = cavernous sinus, SOV = superior ophthalmic vein, ICA = internal carotid artery, IOP = intraocular pressure

## Introduction

Graves-Basedow disease (GBD) is an autoimmune disorder characterized by diffuse goiter, thyrotoxicosis, sometimes associated with other manifestations, such as ophthalmopathy and dermopathy. Graves’ ophthalmopathy (GO) appears in up to 50% of Graves patients. GO’s clinical picture varies in severity and number of manifestations, but it usually appears as a bilateral, often asymmetric orbital inflammation, with a characteristic upper lid retraction. 

Carotid Cavernous Fistulas (CCFs) are abnormal arteriovenous connections between the carotid arteries and cavernous sinus (CS). Clinical findings of CCF include orbital manifestations secondary to the venous congestion, it may be unilateral or bilateral. The symptoms often reported are orbital discomfort, pulsatile proptosis, orbital congestion, conjunctival chemosis, periorbital edema, and ophthalmoplegia, among others. CCF picture can mimic the GO’s manifestations. Thus, CCF should be considered in the differential diagnosis of GO, especially in unilateral, asymmetric, and atypical cases [**1**,**2**]. 

We reported herein a case of CCF in a patient diagnosed with GBD, having a previous history of inactive GO, being a diagnostic challenge.

## Case Report

A 62-year-old female with history of GBD and inactive GO was admitted in the ophthalmology department with progressive exophthalmos in her right eye (RE) for the last two months. One month before, she attended the Emergency Department and was diagnosed with GO reactivation.

**Fig. 1:**
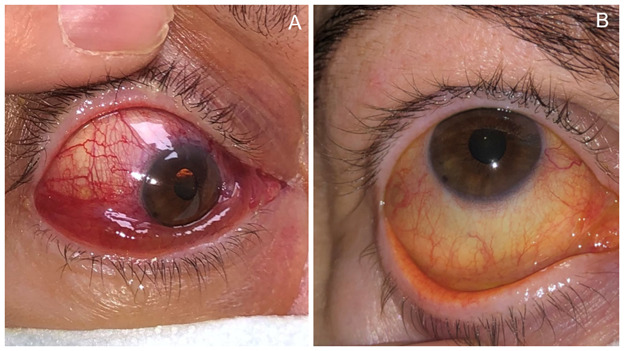
**A.** Slit-lamp photograph (SLP) of the right eye showing chemosis and vascular tortuosity. **B.** SLP: After embolization, chemosis disappeared

On examination, the visual acuity (VA) was 20/ 20 in each eye. Dilated and tortuous conjunctival blood vessels (corkscrew hyperaemia), chemosis and exophthalmos were observed in the RE (**Fig. 1 A**). 

There was an asymmetry in the intraocular pressures (IOP) of 20 mmHg in the RE and 10 mmHg in the LE, both not increasing in up gaze (Bradley maneuver). Extraocular motility and funduscopy were normal. There was no previous history of trauma or high blood pressure. Laboratory results showed normal thyroid function, and positivity of some of the antibodies related to immune thyroid disorders (anti-thyroglobulin and anti-peroxidase). Due to the great asymmetry in the manifestations, which is not common in GO, vascular pathology was included in the differential diagnosis. 

**Fig. 2:**
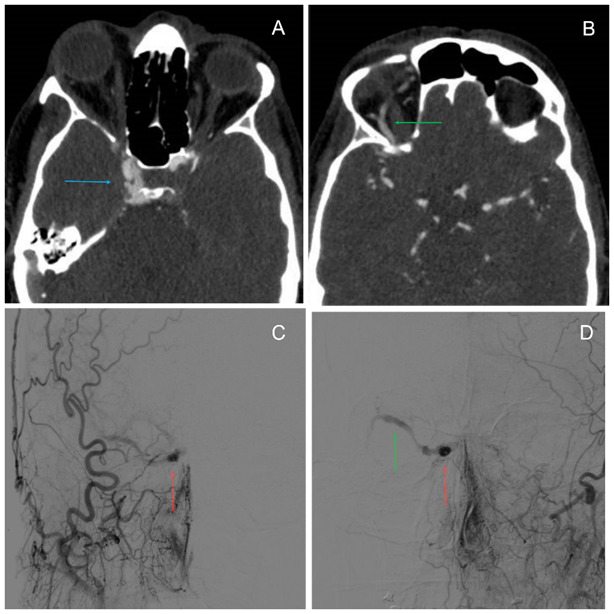
**A.** Angio-CT scan: in the artery phase, an image of early and abnormal filling of the right cavernous sinus (CS) was seen (blue arrow). **B.** Enlarged superior ophthalmic vein (SOV) characteristic of Carotid Cavernous Fistula (CCF) (green arrow). **C** and **D.** Brain arteriography revealed the abnormal filling of the right CS (red arrow) and enlarged SOV (green arrow) from right and left internal carotid artery respectively. Carotid Cavernous Fistula Type B Barrow Classification

Angio-CT scan was performed and showed an early and abnormal filling of the CS and an enlarged superior ophthalmic vein (SOV) (**Fig. 2 A, B**). Brain arteriography confirmed the abnormal filling of the CS from both internal carotid arteries; diagnosis of CCF was established (**Fig. 2 C, D**). Interventional treatment was performed by the neuro-radiologists with good clinical outcome and no recurrence after 6 months of follow-up.

## Discussion

GBD is an autoimmune disorder that mainly affects women, being more frequent between 20 and 50 years of age. GO is the most frequent extra thyroidal manifestation of GBD. Proptosis, orbital congestion, periorbital edema, eyelid retraction, conjunctival chemosis, ocular motility impairment and diplopia are the most common manifestations of GO [**1**,**2**]. Neuroimaging studies have revealed a high number of GBD patients who, despite not having GO symptoms, showed radiological changes such as extraocular muscle involvement [**2**]. Although the most usual cause of orbital inflammation in GBD patients is GO, the physician should take into consideration other possible diagnoses especially in unilateral affectation or very acute onset. Idiopathic orbital inflammation, orbital cellulitis, metastatic tumors, orbital haemorrhage and CCF should be considered in the differential diagnosis of an asymmetric or unilateral eye involvement, even in GBD patients [**1**].

CCF is an abnormal vascular connection between the carotid system and the CS. It can be classified by its aetiology (traumatic or spontaneous, the former being the most frequent), its haemodynamic behavior (high or low flow) and its anatomical arrangement (direct or indirect/ dural) [**3**,**4**]. Barrow classified CCFs in 4 subtypes based on the arterial supply: type A, direct communication between internal carotid artery (ICA) and CS, the most frequent one; type B, communication between dural ICA branches and CS, the present case; type C, communication between dural external carotid artery branches and CS; type D, communication between dural branches of ICA and external carotid artery branches to CS [**3**-**5**]. The dural fistulas usually have low rates of arterial blood flow and may be difficult to diagnose without angiography [**4**]. Pain, pulsatile proptosis, orbital congestion, conjunctival chemosis (corkscrew hyperaemia), periorbital edema, diplopia, and glaucoma are the most common clinical manifestations. The gold standard for the diagnosis is angiography. However, CT and MRI should be the first test to be performed in case of high suspicious. Enlarged SOV and CS and thick extraocular muscles could be detected by these tests [**4**]. However, selective arteriography of carotid arteries may be necessary to completely characterize the blood supply and drainage of cavernous sinus arteriovenous fistula, and for performing selective embolization [**4**].

This clinical case presented as a diagnosis challenge since the first presumed diagnosis in patients with GBD is always GO. Asymmetric eye involvement could be possible in GO but true unilateral disease is uncommon [**2**]. Also, the thyroid-related immune imbalance with some of the antibodies positive could be a confounding factor [**1**]. In addition to the noticeable unilateral inflammation and the type of dilated conjunctival vessels (corkscrew hyperaemia), the engorged SOV and CS did not correspond to a GO and the brain arteriography confirmed the diagnosis of a CCF.

## Conclusion

In conclusion, CCF should be suspected if there is a remarked asymmetry in exophthalmos and IOP, even in patients with GBD, where GO is the most frequent cause of orbital inflammation. In this case, the etiology of the CCF, coinciding with an inactive GO, was unknown. Probably, the increased vascularization, ocular and intracranial pressure changes and pressure on drainage veins could be pathophysiological etiologies in patients with GBD that could lead to the fistula formation, as other authors have suggested. To the best of our knowledge, there are only three previous reports in the medical literature, in which the CCF is diagnosed in a GBD patient with history of GO.

**Conflict of Interest**

The authors declare no conflict of interest.

**Informed Consent and Human and Animal Rights statement**

Informed consent has been obtained from all individuals included in this study.

**Authorization for the use of human subjects**

Ethical approval: The research related to human use complies with all the relevant national regulations, institutional policies, is in accordance with the tenets of the Helsinki Declaration, and has been approved by the Ethics Committee of Virgen de las Nieves University Hospital, Granada, Spain.

**Acknowledgements**

None.

**Sources of Funding**

None.

**Disclosures**

None of the authors has any financial interest to disclose.

## References

[1] Celik O, Buyuktas D, Islak C, Sarici AM, Gundogdu AS (2013). The association of carotid cavernous fistula with Graves’ ophthalmopathy. Indian J Ophthalmol.

[2] Loré F, Polito E, Cerase A, Bracco S, Loffredo A, Pichierri P, Talidis F (2003). Carotid cavernous fistula in a patient with Graves’ ophthalmopathy. J Clin Endocrinol Metab.

[3] Zhu L, Liu B, Zhong J (2018). Post-traumatic right carotid-cavernous fistula resulting in symptoms in the contralateral eye: A case report and literature review. BMC Ophthalmol.

[4] Chaudhry I, Elkhamry S, Al-Rashed W, Bosley T (2009). Carotid cavernous fistula: Ophthalmological implications.. Middle East Afr J Ophthalmol.

[5] Urdapilleta-Contreras M, Padilla-Pérez L, Aguilar-Ruiz A, Verdugo-Unigarro A (2019). Thyroid orbitopathy masked a carotid-cavernous fistula. Case report. Arch la Soc Española Oftalmol (English Ed.).

